# Tourism governance during the COVID-19 pandemic crisis: A proposal for a sustainable model to restore the tourism industry

**DOI:** 10.1007/s10668-021-01707-3

**Published:** 2021-08-29

**Authors:** Rafael Robina-Ramírez, Marcelo Sánchez-Oro Sánchez, Héctor Valentín Jiménez-Naranjo, José Castro-Serrano

**Affiliations:** 1 Department of Business and Sociology, University of Extremadura, Avda de La Universidad S/N, 10071 Cáceres (Extremadura), Spain; 2 Department of Finance and Accounting, University of Extremadura, Avda de La Universidad S/N, 10071 Cáceres (Extremadura), Spain; 3 Department of Arts and Geography, University of Extremadura, Avda de La Universidad S/N, 10071 Cáceres (Extremadura), Spain

**Keywords:** Sustainable tourism, COVID-19, Communities, Governance, Cooperation, Empowerment

## Abstract

Unsustainable models of governance belonging to a widespread neoliberal mindset in developed countries have commonly been applied in the tourism industry. The management of the COVID-19 pandemic crisis has provided exemplary lessons regarding the application of sustainable models of governance. Through a participatory research, guidances are provided to tackle the COVID-19 effects in the tourist sector, namely in the Spanish southwestern region of Sierra de Gata. Seventeen indicators are proposed to enhance the safety measures, commitment of tourist authorities, communities empowered and protection of common resources among tourism industry, tourist authority and communities to spread cooperative awareness, mutual trust and shared objectives. Using a sample of 161 tourism companies, we tested a model of tourism governance with two focus groups during May and October 2020. Structural equation modelling (SEM) was utilized. Based on the data attained from a questionnaire and interviews, a sustainable tourism model to recover the threatened tourism sector is proposed. Indeed, our results can be used to draw theoretical and practical conclusions such as 1.) connecting private and public interactions to tackle the spread of the virus and strategies to recover the damaged tourist sector, 2.) to develop corporative values among the tourist industry and communities, 3.) to enhance governance models (trusts, consortia, tourist boards, clusters) to promote cooperation, 4.) to improve the local participation of companies, communities and associations in decision-making, and 5.) to prioritize qualitative development goals over quantitative ones, in the touristic territory. These conclusions are applicable to other regions suffering from the damaging consequences of the pandemic.

## Introduction

In recent decades, the neoliberal mentality has been spreading in governments and large corporations in developing countries (Seligman and Slobodian, 2020). Neoliberal practices have generated growth and increasing demand based on the promotion of free trade, combined with high global unemployment and the generation of unbalanced wealth (Whelan et al., 2009). Such socioeconomic inequality has reduced the public policies by privatizing state-owned companies and reducing the financing of public services (Allen et al., 2012). These factors have helped weaken governments’ abilities to regulate actions in the public interest (Crotty et al., 1997).

From the liberalist framework, the market must be neutral without affecting the socioeconomic sphere of each citizen, with a deregulating state even in extreme situations such as natural disasters, pandemic crisis, etc. (Harvey, 2005). Under this ideological system, the state must eliminate any intervention in society (Stepney, 2013). Hence, that intervention implies the annihilation of the social protection consciousness (von Sommaruga Howard, 2016), the reduction of social welfare programs and cooperation networks to protect public goods.

Perhaps no service has currently been threatened as significantly as tourism. As Sharma and et al., (2021) has conveyed, millions of jobs in the tourism sector have been put at risk as well as the environmental impact which can be translated in a reduction of touristic activities (Sharma, et al., (2021a). Accordingly, the World Tourism Organization (UNWTO 2020) has claimed that nobody predicted that the pandemic would affect tourism so directly. Moreover, not only it was not on the agenda on previous meetings, but also few of the participants predicted that the tourism sector would be one of the main industries affected (Moreno-Luna et al., 2021).

Since the outbreak of the COVID-19 pandemic, governments, travel agencies and the media and professionals have warned of the contagious risks of the virus in an interconnected world. However, these warnings were not followed in Spain. As a result, it became the second country to have the most cases at one point (Moreno-Luna et al., 2021). Nevertheless, after closing its borders and making repeated announcements to “stay at home,” the disease appears to have been tackled. Close collaboration between the public and private sectors, as a sustainable model, can be replicated from the health sector to other domains, such as tourism.

This investigation aims to transfer this model to the tourism sector through a case study of the tourist areas most seriously devastated by the COVID-19 pandemic. Hence, the following objectives are highlighted as follows: 1.) to establish guidance for the tourist authority to tackle the COVID-19 effects by testing a participatory model among the tourist sector. 2.) According to the first objective, the second proposes to set up touristic indicators based on improving the touristic governance due to COVID-19 pandemic crisis. That governance is based on enhancing; the safety measures, commitment of tourist authorities, empowering communities, protection of common resources. 3.) To spread a high cooperative awareness, mutual trust and shared objectives among the local tourist industry as a way to tackle the liberalism mindset that leads the individual interest and personal gain.

Due to the relevance of the tourism sector, which represents 14% of Spain’s gross domestic product (GDP) (Moreno-Luna et al., 2021), the investigation reflects on way of transferring its health model’s positive effects to the Spanish tourism sector, specifically in the southwestern region of Sierra de Gata. A model of tourism governance was tested in two focus groups during May and June 2020. Based on the literature into models of cooperation between the public and private sectors, seventeen variables were discussed online with 29 companies through two focus groups. As a result of these interactive sessions, all the variables were modified according to the particularities of the tourist destination.

Following this introductory Sect. (1), the structure of this paper is divided into four sections: (2) Material and Methods, where public cooperation models are proposed based on the health governance model derived from the pandemic crisis. A case study site is proposed on the border between Spain and Portugal; (3) Results, obtained from the application of partial least squares structural equation modelling (PLS-SEM) extracted from the companies’ involvement; (4) Discussion based on 25 in-depth interviews and (5) the Conclusions drawn from the Results.

## Literature review

### Sustainable model emerging during the COVID-19 pandemic crisis

Cooperation has been crucial to initiating the recovery process in virus-affected countries (WHO 2020). However, a model of social action cannot be sustainable when it is built on an ideology that merely focuses on developing personal competence through feeding personal interests (Harvey, 2005). According to McCann (2002), liberal ideologies engender unsustainable models of action. They lack the morality to build a society beyond economic interests (Collier, 2011). This is evident when public institutions turn their back on promoting cooperation and meeting the needs of social communities (Amable, 2011).

An alternative economic system is based on cooperation as well as respect for human beings and the environment (Tirole, 2017). In such a cooperative model, individuals are able to sacrifice personal benefits to build a common good (Nowak & Highfield, 2011). For instance, during the COVID-19 crisis, professionals from a range of sectors such as supermarket workers, elderly carers, teachers and social workers have carried out essential and challenging tasks (Williamson et al., 2020). Similarly, the underestimated workforce of nurses has come to occupy frontline positions working with stress and anxiety, with little protection against possible infections (Jackson et al., 2020).

By working together, the workforce has engaged in a manner inconsistent with the innate tendency of selfish survival, putting individual prosperity at the service of greater common good and recovering social and cooperative values amazingly absent in liberal ideology. This cooperative model has multiplied the public services, promoting collaboration between citizens, organizations and governments to curb the transmission of the virus (Rizzi et al., 2018). According to Vargas (2020, p. 2), it is necessary an effective integration and active participation of people who live in the destination to achieve a sustainable model. It leads the industry to be oriented toward education, environmental and social justice. Tourists, local communities, SMEs, Government need to take advantage of the devastating pandemic situation (Sharma et al., 2021). That model reinforces the idea of tourism destination as an interdependent business ecosystem, capable of anticipating outcomes through data-driven and technology developments.

According to the main tourist organization in Spain, called Hosteltur (2020), a cooperative model has existed since the beginning of the crisis, but without success. In response, touristic institution has established a sustainable model of public–private collaboration between tourism companies and local, regional and national tourism authorities.

### Commitment of tourist authorities to restoring the image of the tourist destination

In the work “Regenerative tourism needs diverse economic practices,” Cave and Dredge (2020) propose that a new relationship between tourism and capitalism must be reshaped as a result of COVID-19. New pathways to provide more resilient and regenerative tourism practices should replace the damaging capitalism’s emphasis on resource exploitation, growth and profit based on selfishness and personal interest.

One of the main challenges facing tourism authorities is to invest in human capital in order to rebuild a tourism industry threatened by the pandemic. According to Goméz et al. (2013), the authority’s commitment to restoring tourism should be focused on ascertaining the risks of not providing the necessary safety to visitors and on promoting strategies that involve tourist agents in the process of rebuilding that safety. Hence, the objective of restoring tourism would begin by analyzing the perception of safety that communities have in the construction of the image of the destination until a situation of “no risk perception” for tourists has been reached (Hosteltur, 2020).

To build a “safe” image of a tourist destination, close collaboration must be developed between communities, authorities and companies. Higgins-Desbiolles (2008) has highlighted three levels of reforms: (a) mild, through the introduction of corporate social responsibility (CSR) policies in companies; (b) intermediate reforms, applying the consequences of fair trade between tourists, tourism-receiving communities and public authorities; and (c) the application of a humanistic vision of tourism management, which involves all tourist agents. This new vision incorporates models of good practice associated with participatory and community tourism (Sánchez-Oro Sánchez and Robina-Ramírez 2020). The third reform would allow the touristic authorities to promote justice awareness among communities and small business by eliminating bad practices by tourist authorities and large corporations, thereby rebuilding society based on socialization processes.

Scott (2006) has defined such socialization processes as the integration of tourism in the recovery process of destinations from the determination of needs to the definition of the minimum parameters of their citizens’ well-being. These parameters also include the risks associated with health during the pandemic crisis, especially in terms of infectious diseases among foreign tourists (Wong et al., 2020).

In Spain, very few studies have incorporated the variable of safety as part of a tourist destination. However, Gómez et al. (2013) have studied the image of inland destinations from the perspective of residents and visitors in Spanish regions such as La Rioja, Castilla y León, Extremadura and Castilla La Mancha. This work refers to Beerli and Martín (2004) by mentioning the dimension of social conditions, safety, cleanliness and hospitality. Indeed, these social determining factors were found to decisively influence “the tourist experience” both for visitors and the local population (Goméz et al. 2013 p. 170). Moreover, studies carried out in the Extremadura region, prior to the pandemic crisis, have confirmed the safety of the destination from a health point of view (San Martín Gutiérrez and Rodríguez del Bosque, 2010), with clean and neat tourist areas (Luque et al. 2007), friendly and hospitable inhabitants (Curtin, 2010), a pleasant climate (Beerli and Martín, 2004) and spaces free of environmental contamination (Echtner, 1991). The development of a strategy to recover this image of safety today is going ahead. As a result, several hypotheses are proposed here, the first two being:

#### Hypothesis 1 (H1)

The commitment of tourist authorities (CTA) to restoring the image of the tourist destination facilitates the adoption of safety measures (SM) therein.

#### Hypothesis 2 (H2): 

The implementation of safety measures (SM) in the tourist destination facilitates sustainable models of tourist governance (SMTG).

### Empowerment of tourism recipients to develop tourist destinations

The recovery of the image of the destination and the integration of tourism in society entails empowering the communities, companies and local associations that receive tourism through inclusive, fair and equitable strategies (Scheyvens, 2003). According to Benjamin, Dillette, & Alderman (2020), a resilient post-pandemic tourism should be more just and equitable based on sustainable operations but also how ethics is leading tourist practices and decisions from the tourism industry and authority. It requires stepping away from selfish perspective to understand the tourism as a valuable resource to preserve. As an example from the COVID-19 crisis, health managers and communities have worked cooperatively, requiring public authorities’ commitment, transparency and involvement.

From this point of view, tourist authorities must not only include the relevant communities, companies and associations in tourism planning, but also integrate tourists in the responsible tourism strategy. New strategies should delve into new ways of connecting people while respecting the social/physical distancing. According to Lew, Cheer, Haywood, Brouder & Salazar (2020), a resilience adaptative actions must lead the developing of tourist destinations to avoid the touristic system’s collapse: (1) Innovation and creativity based on a new-organizations of the touristic resources, (2) channel appropriately and responsibly the touristic opportunities to adjust the growth according to the planet, (3) implement rules by institutions to consolidate the tourism´s sustainable awareness.

In the tourism sector, such cooperative work contains five aspects: (1) tourist facilities as a key element of tourist planning and development of destinations, providing solutions from the tourist authorities to seasonality and infrastructure maintenance costs (Scheyvens, 2003); (2) the provision of emergency health services to control the effects of the pandemic and new outbreaks of the virus (Horowitz, 2007) as well as other financial and economic services to favor the tourism industry for the benefit of local entrepreneurs; (3) training communities to develop cooperative work for the maintenance of tourist facilities (Lucchetti & Font, 2013); (4) the improvement of transport infrastructure to support tourism and local development, especially in remote destinations, by solving the mismatch between tourism demand and supply (Gössling et al., 2020); and 5) build social justice related to the distribution of costs and benefits at the local, regional and national level (Ashley et al., 2000). These five aspects make it possible to build safer tourist destinations and to manage them during the pandemic crisis and the post-virus phase with greater guarantees of success.

Cooperative work empowers communities not only to maintain the tourist facilities but to raise emotional involvement for tourists and companies through “human flourishing.” It releases positive emotions, provides a sense of achievement through pursuing purposeful goals, builds meaningful relationships and energizing stakeholders to tackle the damaging effect of COVID-19 pandemic (Cheer, 2020).

The importance of community empowerment in the management of tourism crises leads us to suggest the following hypotheses:

#### Hypothesis 3 (H3)

The commitment of tourist authorities (CTA) to restoring the image of the tourist destination facilitates the empowerment of local communities (ECT).

#### Hypothesis 4 (H4)

The empowerment of local communities (ECT) facilitates sustainable models of tourism governance (SMTG).

#### Hypothesis 5 (H5)

The empowerment of local communities (ECT) facilitates the implementation of safety measures (SM) in the tourist destination.

### Respect for common resources in the tourism sector

The protection of resources with the attributes of tourism products (e.g., water resources, ecosystems, forests and oceans) located in public spaces (Briassoulis, 2002) is subjected to variable demand. According to neoliberal ideology, the protection of common goods for citizens has commonly come into question (Dahl & Soss, 2014). However, in the United Kingdom, as an example of a liberal country, the cooperative attitude of many British citizens in defending a common good such as health has led the neoliberal prime minister to publicly acknowledge the “existence of society” (Johnson, 2020).

Tourism, conceived as a transformative ideology at the service of controlling of the economy and power (Tribe, 2006), must be built on a basis of cooperation, especially between receiving communities (Desmond, 1999).

The question is to investigate whether under this new perspective of the post-crisis era it is possible to develop tourism with a human face, which takes into consideration the receiving communities and the impacts caused by tourist flows (Sömmez, 2002).

During the pandemic, cooperation policies combined with safety measures were implemented to save individuals. Equally, during the ongoing tourism crisis, common good should also be protected by developing cooperation policies to distribute wealth equally among affected communities in tourist destinations (Jamal et al., 2013).

In this sense, research into inequality and tourism (Cole & Morgan, 2010) as well as fair tourism (Higgins-Desbiolles, 2008) have raised questions about who benefits from tourism, how tourism can become more just, what the purposes of tourism are and how tourism can become a tool to guarantee justice (Higgins-Desbiolles, 2018; UNWTO, 1999). The need to respect common tourism resources in such a fair, cooperative scenario leads us to pose the following hypotheses:

Hypothesis 6 (H6): The protection of tourist resources (PTR) facilitates sustainable models of tourism governance (SMTG).

Hypothesis 7 (H7): The introduction of safety measures (SM) in the tourist destination facilitates the protection of tourist resources (PTR).

Hypothesis 8 (H8): The commitment of tourist authorities (CTA) to restoring the image of the tourist destination facilitates the protection of tourist resources (PTR).

## Dada and method

### Case study

**Fig. 1:**
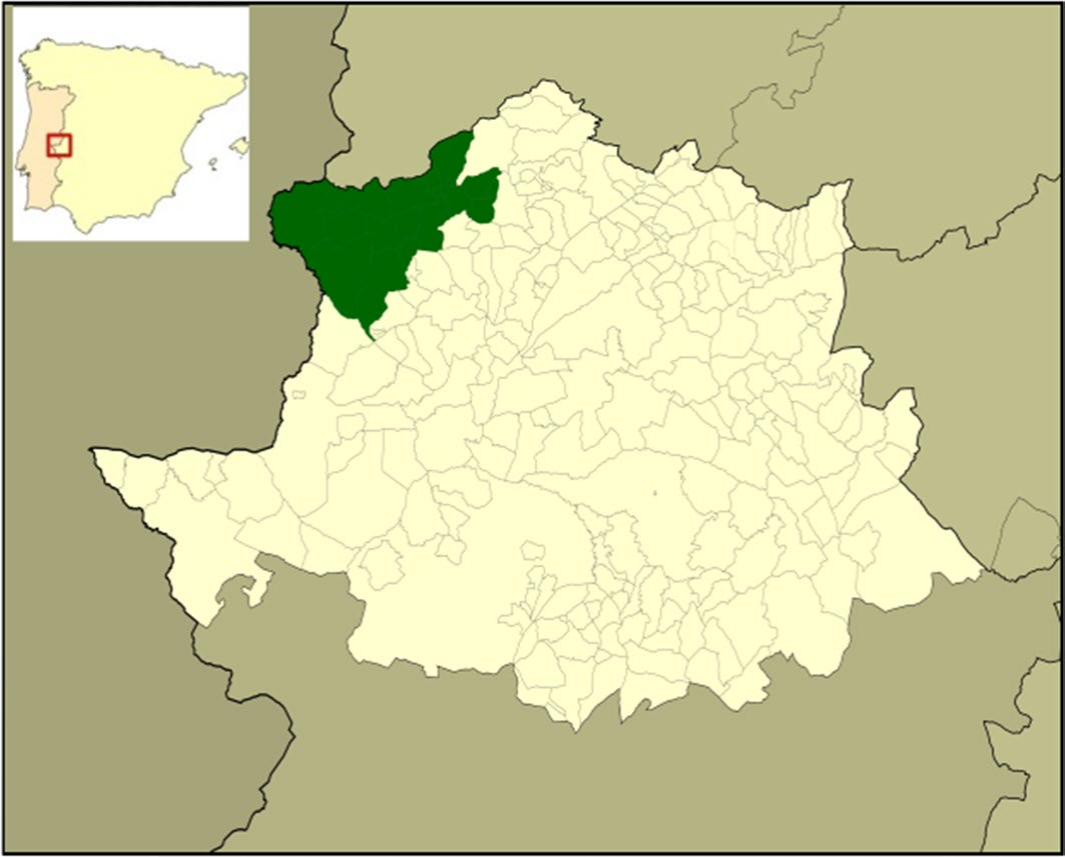
Location of Sierra de Gata (Cáceres)

The Sierra de Gata region has become one of the most threatened tourist areas in Spain due to its relative inaccessibility. It is located in the extreme northwest of the autonomous community of Extremadura, in the province of Cáceres, on the border between Spain and Portugal. It has a population of approximately 25,000 inhabitants distributed across 19 municipalities. The number of tourists in 2017 multiplied that figure by three (Adisgata, 2017) (see Fig. 1).

Tourism to this region was blocked by the decree of the Spanish government preventing movement between provinces for more than three months (Royal Decree-Law 16/2020).

The area is surrounded by mountains. It cannot be accessed by train, plane or highway. This has rendered the space a natural reserve, celebrated for the diversity and beauty of its flora, fauna and hydrographic resources. As a result, it is currently one of the areas with the greatest potential and growth for the practice of inland tourism. According to published studies (Adisgata, 2017), 84.5% of visitors to the destination choose it as a resting space. Moreover, 77.9% of its visitors aim to learn about the historical-artistic heritage of the area, 69.7% seek to experience it as a natural space and 59.9% intend to enjoy the local gastronomy.

In total, 91.3% of its visitors are national while 8.7% come from abroad. Portugal provides the highest share of visitors, despite the fact that visit records have decreased by more than 10 percentage points. Within the Spanish group, the majority come from Madrid.

## Research design

Data from tourism companies were provided by the three tourist offices in the area. The number of tourism companies and places available in the tourist region is shown in Table 1.

**Table 1:** Population and sample

Tourism companies	Number	Beds
Tourism activity companies	17	26
Cottages	4	56
Rural apartments	24	247
Apartments in villages	1	5
Rural houses	44	363
Lodging houses	14	217
Hotels	3	74
Pensions	1	24
Bed and Breakfast	4	81
Camping sites	10	991
Restaurants	39	–
Total	161	2084

*Source*: ADISGATA, Cáceres 2017—Provincial tourist Association. Retrieved from http://www.sierradegata.org/turismo/ocio/ARbuscar.asp

To measure the 161 tourism companies’ perceptions of potential sustainable tourism management models, the research team contacted them by email. Seventy-eight (almost 50%) responded with interest to participate in the research. The remainder were contacted by phone. Five decided not to participate in the investigation.

At the beginning of the study, the companies were asked whether they would prefer to participate in two focus groups through the Zoom online platform. A total of 29 companies responded affirmatively. The focus groups were organized according to two types of tasks: 1.) To discuss the main current challenges in the tourism sector under the COVID-19 crisis and their recovery relations to the local tourist destination, and 2.) to test the indicators extracted from the literature review and the measures proposed by the tourism companies with the aim of adapting them to the reality of tourism in the area. The indicators were also re-grouped into constructs.

### Variables and questionnaire

As a result of the interactive meetings, five variables and 17 indicators were chosen. Fifteen of the 17 were modified by the tourism companies in the previous online session (see Table 2).

**Table 2:** Preliminary study and list of items corrected by the managers

Originally proposed items	Corrected items
SMTG1: To manage tourism destinations, it is necessary to move away from neoliberal principles built on individual success toward principles of collaboration between communities and local authorities and companies (Nelson, 2007)	SMTG1: Due to the serious tourism crisis in our region, the sector requires establishing collaborative protocols between companies, tourism authorities and communities (Nelson, 2007)
SMTG2: It is possible to apply the citizen collaboration that has existed to stop the pandemic to tourism in order to improve the tourist destination through cooperation (Robina-Ramírez et al., 2021)	SMTG2: Citizen collaboration is vital to stop the pandemic and bring tourism to the area (Robina-Ramírez et al., 2021)
SMTG3: Cooperation between authorities and communities in the destination should enable proposals, attitudes and behaviors to be adapted to that objective (Sánchez et al., 2021)	SMTG3: Tourism authorities should be involved in designing a plan for the economy (Sánchez et al., 2021)
PTR1: The purposes of tourism must go beyond a mere economic vision and instead define and develop tourism resources for all tourism agents (Tribe, 2006)	PTR1: Companies and communities must rebuild tourism and the economy by working together (Tribe, 2006)
PTR2: The protection of common assets and resources requires the active participation of all the social agents in the destination (Hess, 2008, Oro et al., 2021; Ostrom, 1990)	PTR2: Protocols for the protection of tourism resources should be periodically assessed (Hess, 2008, Oro et al., 2021; Ostrom, 1990)
PTR3: The design of incentives by local authorities is key to maintaining or improving resources in a balanced way, avoiding any overexploitation (Healy, 1994)	PTR3: Incentives to improve the quality of training play a key role in this pandemic crisis (Healy, 1994)
PTR4: It is necessary to plan the equitable distribution of the benefits of resource management among tourism-receiving communities (Cole & Morgan, 2010; Higgins-Desbiolles, 2008; Jamal et al., 2013)	PTR4: Equitable distribution of benefits in the tourist destination should be implemented (Cole & Morgan, 2010; Higgins-Desbiolles, 2008; Jamal et al., 2013)
CTA1: The incorporation of CSR and fair trade measures in tourism management by companies and local authorities would help to restore the tourist destination (Higgins-Desbiolles, 2008)	CTA1: CSR measures are important to restore the image of the destination (Higgins-Desbiolles, 2008)
CTA2: The authorities in the region should be more committed to being part of the tourism image development plan (Sánchez-Oro Sánchez & Robina-Ramírez, 2020)	CTA2: All tourism agents should be involved in building the destination’s image (Sánchez-Oro Sánchez & Robina-Ramírez, 2020)
CTA3: The integration of tourism in society requires that the socioeconomic and tourist needs of the destination be planned with all the social actors (Scott, 2006)	CTA3: The tourism plan should include visitors to the destination (Scott, 2006)
ECT1: The empowerment of the tourism sector requires a proposal for a consensual improvement of tourist facilities in the destination (Scheyvens, 2003)	ECT1: Companies should be empowered by obtaining decision-making capacity regarding the public budget (Scheyvens, 2003)
ECT2: Empowering tourism-receiving communities in the current pandemic entails the provision of health services in the destination (Horowitz, 2007)	ECT2: Empowering communities means to attain knowledge about the health protection measures program (Horowitz, 2007)
ECT3: Developing a destination requires improvements to transport infrastructure in accordance with local development (Fallon & Kriwoken, 2003; Gössling et al., 2020)	ECT3: Communities need to be made familiar with the infrastructure plan to make investments in the future (Fallon & Kriwoken, 2003; Gössling et al., 2020)
ECT4: Elements of social justice related to the distribution of the costs and benefits generated by destinations should be incorporated (Ashley et al., 2000)	ECT4: Transparency regarding the benefits and costs of tourism in the region is important (Ashley et al., 2000)
SM1: Health safety protocols must be established throughout the tourist destination (San Martín Gutiérrez & Rodríguez del Bosque, 2010)	SM1: Health safety protocols should be established throughout the tourist destination (San Martín Gutiérrez & Rodríguez del Bosque, 2010)
SM2: Tourist areas must be clean and neat (Luque et al., 2007) and environmental pollution (e.g., traffic, smoke, noise) low (Echtner, 1991)	SM2: Monthly control mechanisms should be formalized so that the destination and its resources can be cleaned and advertised (Echtner, 1991)
SM3: The pleasant climate of Extremadura helps tourist to enjoy the tourist destination (Beerli & Martín, 2004)	SM3: The microclimate of the region, especially in summer, should be publicized to attract tourism (Beerli & Martín, 2004)
SM4: The friendly and hospitable inhabitants in Extremadura move tourists to return to the destiny (San Martín Gutiérrez & Rodríguez del Bosque, 2010)	SM4: The destination should be advertised to customers on regional and national tourism portals (San Martín Gutiérrez & Rodríguez del Bosque, 2010)

*Own source*

**Fig. 2:**
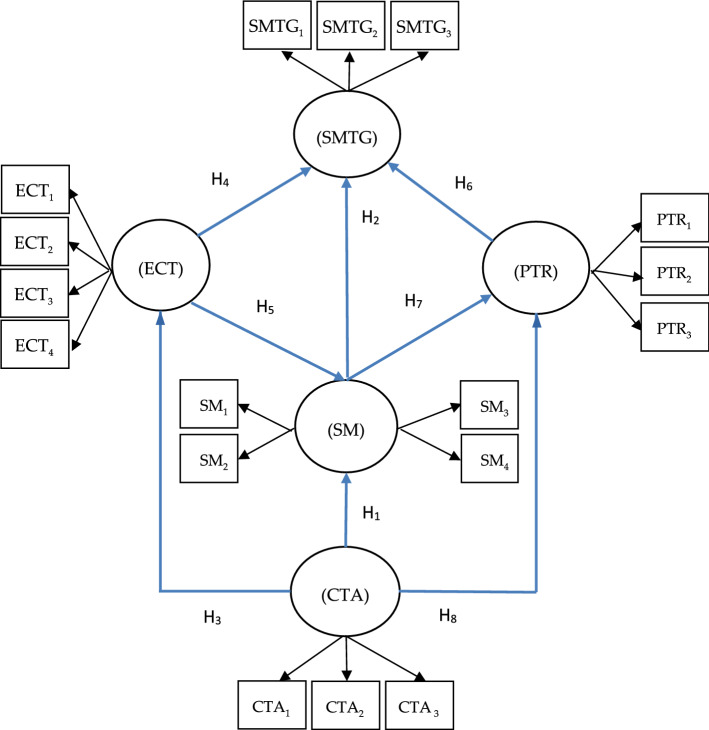
Model

The five latent variables were connected and are displayed in Fig. 2 according to the meaning of each variable and its relationship with the others. Then, in order to develop sustainable models of tourism governance for the recovery of tourism in Extremadura (SMTG), four variables were defined. Tourism governance starts with defining (in the current COVID-19 pandemic) the commitment of tourist authorities to restoring the image of the tourist destination (CTA). Such commitment is directly related to the adoption of safety measures in the tourist destination (SM). Installing safety measures is not solely dependent on the tourist authority’s side, but also on the community side. Subsequently, cooperation models based on empowering communities (ECT) need to be promoted. Similarly, safety measures can protect tourism’s public goods and resources (PTR).

Each item was formulated in question mode. The questionnaire was previously validated through 15 qualitative interviews conducted with hotel managers, who differed from the participants in the two focus groups.

According to the model, the hypotheses are as the following:

#### Hypothesis 1 (H1)

The commitment of tourist authorities (CTA) to restoring the image of the tourist destination facilitates the adoption of safety measures (SM) therein.

#### Hypothesis 2 (H2)

The implementation of safety measures (SM) in the tourist destination facilitates sustainable models of tourist governance (SMTG).

#### Hypothesis 3 (H3)

The commitment of tourist authorities (CTA) to restoring the image of the tourist destination facilitates the empowerment of local communities (ECT).

#### Hypothesis 4 (H4)

The empowerment of local communities (ECT) facilitates sustainable models of tourism governance (SMTG).

#### Hypothesis 5 (H5)

The empowerment of local communities (ECT) facilitates the implementation of safety measures (SM) in the tourist destination.

#### Hypothesis 6 (H6)

The protection of tourist resources (PTR) facilitates sustainable models of tourism governance (SMTG).

#### Hypothesis 7 (H7)

The introduction of safety measures (SM) in the tourist destination facilitates the protection of tourist resources (PTR).

#### Hypothesis 8 (H8)

The commitment of tourist authorities (CTA) to restoring the image of the tourist destination facilitates the protection of tourist resources (PTR).

## Results

### Measurement model results

The information obtained was processed following the parameters of SEM. This statistical technique is used when dependency relationships are established between latent variables and indicators (Sarstedt et al., 2016). For the generation of the statistical model, the PLS technique applied was SmartPLS 3, Version 26.

PLS-SEM was defined based on two approaches: the measurement model and the structural model. To proceed to the analysis of the structural model, it was necessary to analyze the reliability existing between the indicators and the constructions as well as the validity of the measurement model (Hair et al., 2016). To this end, we used reflective elements, because they are interchangeable. Reliability was studied by analyzing individual loads or simple correlations of the measures with their respective latent variables (load ≥ 0.7; Henseler & Ringle, 2009). Of the 17 variables, two were rejected (SM1; ECT1) because their loads were lower than 0.7.

Cronbach’s alpha and composite reliability were used as the reliability indexes of the latent variables. The convergent validity of the latent variables were analyzed through the extracted average variance (AVE) (accepted > 0.5). To study the discriminant validity of the latent variables, the Fornell-Larcker criterion was used (Fornell & Bookstein, 1982). This criterion examines whether the square root of the extracted average value (AVE) of each item is greater than the correlations with the other latent variables, as shown in Table 3.

**Table 3:** Reliability, validity of the constructs

Statistics	Cronbach Alfa	rho_A	CR	AVE	Fornell-Larcker Criterion				
Variables					PTR	SM	SMTG	CTA	ECT
PTR	0.800	0.808	0.882	0.713	0.845				
SM	0.876	0.878	0.924	0.802	0.439	0.895			
SMTG	0.870	0.881	0.920	0.794	0.663	0.645	0.891		
CTA	0.900	0.902	0.938	0.834	0.435	0.404	0.514	0.913	
ECT	0.813	0.819	0.889	0.727	0.461	0.730	0.630	0.512	0.853

*Own source*

According to Henseler et al., (2015), it is necessary to implement techniques that better detect the absence of discriminant validity. In this case, the test applied is called the heterotrait-monotrait ratio (HTMT). If the ratio for each pair of factors is < 0.90, the condition is accepted (Henseler, 2017). Table 4 shows the valid values for the HTMT test.

**Table 4:** Heterotrait-monotrait ratio (HTMT)

	PTR	SM	SMTG	CTA	ECT
PTR					
SM	0.513				
SMTG	0.779	0.737			
CTA	0.509	0.454	0.579		
ECT	0.557	0.864	0.727	0.592	

*Own source*

### Results of the structural model

After examining the measurement model, we moved on to the structural model. For this, the path coefficients of each of the hypotheses were studied. To obtain these values, a 5000 subsample start-up program was applied to verify the statistical significance of each path.

The general fit of the model was evaluated using various indicators (see Table 5). We started with the standardized root-mean-square residual (SRMR) indicator, which is the average difference between the predicted variations and the covariance and those observed in the model (Hu & Bentler, 1998). A value < 0.8 reflects a good fit in the estimated model (Henseler & Ringle, 2009). In our case, the value was 0.076 and so it was accepted. Bentler-Bonett Normed Fit Index (NFI) values were used. NFI values vary between 0 and 1, with the closer to 1, the better the adjustment. In our case, the values were very close to 1, indicating a good fit. The RMS_theta measures the degree of correlation of the residuals of the external model (Wong et al., 2020). Values close to zero indicate a good fit, which was also true of the extracted data.

**Table 5:** Model fit

	Saturated model	Estimated model
SRMR	0.073	0.076
d_ULS	0.631	0.694
d_G	0.410	0.415
Chi-cuadrado	299.319	299.026
NFI	0.877	0.877

*Own source*

The explained variance (*R*^2^) of the endogenous latent variables and the p value of the regression coefficients (t test) were used as indicators of the explanatory power of the model. The level of significance is determined from the nonparametric value of the t-distribution derived from the re-sampling or bootstrapping process. When in a model the hypotheses indicate the relation of the direction (+ or –), it is necessary to use a one-tailed t-distribution with *n*-degrees of freedom, where n refers to the number of subsamples (bootstrapping = 5,000 subsamples; *t* = 0.1; 4,999 = 1,645; *t* = 0.05; 4,999 = 1,960; *t* = 0.01; 4,999 = 2,577; *t* = 0.001; 4,999 = 3,292). Statistical significance can also be verified from *p* values * *p* < 0.05; ** *p* < 0.01; *** *p* < 0.001 (Wong et al., 2020) (see Table 6).

**Table 6:** Path coefficients

Statistics/ Variables	*β*	Lower CI	Higher CI	*t* Statistic	*P* value
H1: CTA → SM	0.041	− 0.069	0.166	0.696	0.487 (ns)
H2: SM → SMTG	0.300	0.300	0.167	4.450	0.000***
H3: CTA → ELC	0.512	0.517	0.368	7.029	0.000***
H4: ECT → SMTG	0.211	0.069	0.357	2.855	0.004*
H5: ECT → SM	0.709	0.589	0.810	12.542	0.000***
H6: PTR → SMTG	0.434	0.272	0.575	5.564	0.000***
H7: SM → PTR	0.315	0.157	0.466	3.991	0.000***
H8: CTA → PTR	0.308	0.117	0.491	3.155	0.002**

*Own source.* Statistical significance: *p < 0.05; **p < 0.01; ***p < 0.001; n.s: not significant

Geisser (1974) and Stone (1974) have recommended the Stone–Geisser test as a criterion for evaluating the predictive capacity of the model (*Q*^2^). To determine this in SmartPLS, one must undergo the blindfold procedure. Following the Stone–Geisser (*Q*^2^) test (Geisser, 1974; Stone, 1974), the values were 0.02, 0.15, and 0.35, indicating small, medium, and high predictive relevance, respectively. As a result, Table 6 shows that the endogenous constructions were (*Q*^2^) > 0. The values of R2 maximized the amount of explained variance obtained for the investigation and led to the following conclusions: 0.67 “Substantial,” 0.33 “Moderate” and 0.19 “Weak” (Chin, 1998).

We can say that the proposed sustainable model of cooperation (SMTG) for the recovery of the chosen tourist destination was significant, with a moderate explanatory capacity (*R*^2^ = 61.5%). This means that the selected variables CTA, SM, ECT and PTR explained 61.5% of the proposed model and represent key factors for the development of sustainable models of cooperation for tourism recovery (see Table 7).

**Table 7:** Coefficient of determination (*R*^2^) and Stone-Geisser test (*Q*^2^)

Variables	*Q*^2^	*R*^2^
PTR	0.178	0.272
SM	0.422	0.534
SMTG	0.473	0.614
CTA		–
ECT	0.175	0.256

*Own source*

In addition to evaluating the *R*^2^ value of all endogenous constructs, it was necessary to ascertain the change in *R*^2^ when a certain exogenous construct was omitted from the model. In such a case, the *f*^2^ might be used to assess whether the omitted construct has a substantive impact on endogenous constructs. To this end, Cohen (1998) has specified the following values to evaluate the *f*^2^: 0.02 is a small effect, 0.15 is a moderate effect and 0.35 is a large effect. As Table 8 shows, the main effect (*f*^2^ = 0.796) refers to the strong effect that the empowerment of tourism-receiving communities (ECT) has in the safety measures implemented in the destination (SM).

**Table 8:** Effect size

	PTR	SM	SMTG	CTA	ECT
PTR			0.373		
SM	0.114		0.106		
SMTG					
CTA	0.109	0.003			0.356
ECT		0.796	0.051		

*Own source*

Two other relationships with an important effect identified were, on the one hand, the protection of common goods and tourist resources (PTR) is strongly related to sustainable models of tourism governance (SMTG) (*f*^2^ = 0.373) and, on the other, the commitment of tourist authorities to restoring the image of the tourist destination (TAC) is greatly affected by the empowerment of tourism-receiving communities (ECT) (*f*^2^ = 0.356).

**Fig. 3:**
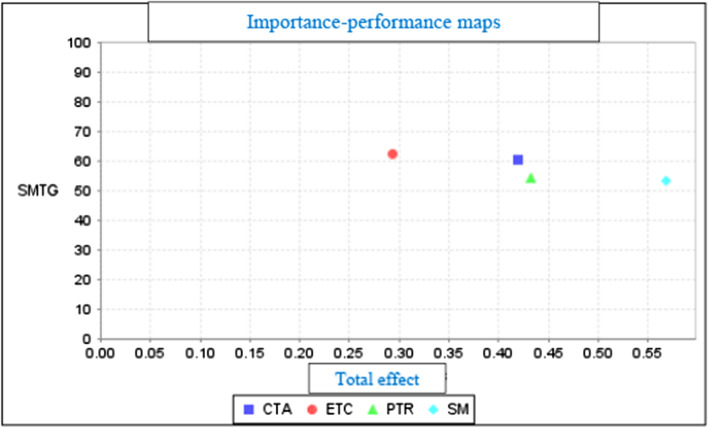
Importance-performance maps of constructs

According to research that studied data heterogeneity the IPMA (Internet Search Engines), PLS technique was used to find more precise recommendations for marketing of Internet search engines (Woodside, 2013). IPMA is a framework study that uses matrices that enable combining the average value score for “performance” with the estimation “importance” in PLS-SEM’s total effects (Woodside, 2013). The outcomes are shown through the importance-performance chart. According to Fig. 3, the construct ETC (62.521) has higher performance in relation to SMTG than the other constructs: CTA (60,598), PTR (54.474) and SM (53.409).

**Fig. 4:**
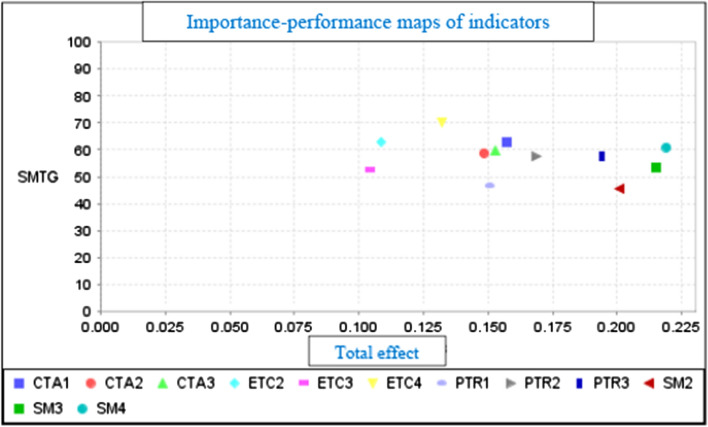
Importance-performance maps of indicators

Figure 4 shows the performance of each indicator in relation to SMTG. Indicators ETC4 (70,464), ETC2 (63,038), and CTA1 (62,814) have the highest values in comparison to the other indicators.

## Discussion

The governance of the COVID-19 pandemic crisis has demonstrated the importance of having committed health authorities and a highly involved citizenry to face the challenge of stopping the spread of the virus. A new form of cooperative awareness has emerged as a consequence of the health crisis. Due to the COVID-19 outbreak, the tourism industry has been hit hard internationally, especially in regions with poor communication access, such as the case study presented in this paper.

The considerable significance of the model of tourist governance we have proposed is corroborated by the fulfilment of most of our hypotheses. It is interesting to observe how the commitment of tourist authorities to restoring the image of the tourist destination (CTA) is channelled not only through the adoption of safety measures in the tourist destination (SM) (H1: CTA → SM; *β* = 0.041; *T* = 0.696) but through the empowerment of tourism-receiving communities (ECT) (H3: CTA → ECT; *β* = 0.512; *T* = 7.209) and the protection of common goods and tourist resources (PTR) (H8: CTA → PTR; *β* = 0.308; *T* = 3.155).

These theses have already been suggested by Scheyvens (2003), who argues that the integration of tourism in society entails empowering the communities, companies and local associations that receive tourism through inclusive, fair and equitable strategies, including the protection of the resources of tourist destinations. Therefore, the commitment of tourist authorities to restoring the image of the tourist destination (TAC) is not an activity that must be carried out unilaterally by the tourist authority, but rather involves encouraging the protection of tourist resources and equitably distributing the benefits of resource management between recipient communities and local companies (Cole & Morgan, 2010; Higgins-Desbiolles, 2008; Jamal et al., 2013).

The interviews corroborated these results. Some managers emphasized that “public–private collaboration is now more necessary not only in the tourism sector but among all the economic actors in the region” (Interview 7). Interview 14 reveal that “to overcome a health crisis, a strategy need to be built based on strengthening the relations between competitors: those who compete for tourist spending must now cooperate to restore tourist confidence” (Interview 14). This cooperative attitude, akin to that used in the fight against COVID-19, was also perceived in other interviews: “mutual trust and collective thinking are the basis for renewed post-COVID-19 tourism development” (Interview 12).

This cooperative attitude currently underlies the minds of many entrepreneurs: “it is necessary to create a joint value, having shared objectives to rebuild tourism” (Interviews 1, 2, 5, 8, 10); “this work will require unprecedented time and effort to develop tourism industry free of COVID-19” (Interview 17). Within the shared objectives, several entrepreneurs highlighted “the importance of developing safety protocols with plans and warning and reaction systems in case of need, even if they never have to be used” (Interview 3). Moreover, “it is not only important to have protocols but also to publicize them” (Interviews 4, 9 and 15).

According to the data, the proposed sustainable cooperation model (SMTG) (*R*^2^ = 61.5%) incorporates the concerns of the entrepreneurs in the area. Not only did the respondents suggest that the greater involvement of tourism authorities (CTA) is necessary to developing safety protocols (SM), but also that empowered communities (ECT) are desired to restore and protect tourist destinations.

The development of safety protocols currently plays a key role among tourist authorities and communities to recover the tourist destiny in the case study. Indeed, the companies suggested that it is important to “configure the tourist destination as if it were a large company, setting a participatory planning process with the complicity and active collaboration of residents” (Interviews 16 and 19) and “within the joint actions would be the processes of tourism innovation and marketing” (Interview 20).

## Conclusions

The widespread neoliberal mindset in large corporations and governments around the world has become inefficient to face unexpected challenges of society. Natural disasters, the pandemic crisis, to mention but a few, do actually demand collaborative strategies and cooperative actions to restore what has been damaged.

Since the outbreak of the COVID-19 pandemic, a large amount of cooperative work has been carried out on families, communities, countries and continents. Even neoliberal governments have recognized the role that society plays in reducing the unexpected effects of the pandemic crisis.

Should those coordinated effects be transferred into the touristic sector, several actions will be needed to be deployed in order to minimize the devastating impact on the tourism industry. A sustainable model of tourist governance is proposed in this research work after having tested one hundred and fifty-six Spanish companies from different underprivileged areas throughout Spain. This model is based on what we have witnessed in the health sector over the last two years.

Policy recommendations and practical implications can be drawn from the model proposed in the research work, which is organized in three areas: personal training, defining regenerative tourism practices to protect touristic businesses and resources and collaborative processes with touristic authorities.

### Personal training to restore the damaged touristic sector

Training sections are key to promote the common good as to restore trust in the touristic sector. Instead of promoting neoliberalism interests regarding the management of touristic businesses, practical implications should be drawn by putting individual prosperity at the service of greater common good. First of all, it is deemed urgent to recover social and cooperative work amazingly absent in liberal ideology. Secondly, as it has happened in the health sector, the promotion of educational tourism and the development of citizen awareness, social tourism and cooperative exchanges between geographically adjacent tourist communities then become crucial. Thirdly, in order to achieve that purpose, training sessions from a humanistic perspective could analyze devastating effects for both touristic companies and communities with the aim to eradicate the exploitation and growth mindset based on selfishness and personal interests. Hence, that humanistic vision is built over personal values and basic moral principles which may influence personal conducts to achieve common goods (Haste, 2010).

### Resilient and regenerative tourism practices

Considering the devastating effects of the pandemic, only collaborative private and public plans for local tourism communities and companies can help restore what has been lost. Natural resources and jobs related to the tourist sector can be protected by creating collective actions. The connection between touristic authorities, companies and residents plays a key role in regenerative tourism practices (Amable, 2011; Higgins-Desbiolles, 2008). Those regenerative touristic practices are part of a long-term plans based on four elements: 1.) the definition of minimum parameters regarding the well-being of citizens 2.) the protection of touristic resources in specific tourism destinations, 3.) the risks associated with health during the pandemic crisis, and 4.) the economic recovery process of local tourism based on business reduction of taxes, recovery business plans, etc. (Wong et al., 2020).

As a common issue, the resilient practices designed for the authorities at the tourist destination must include healthy parameters such as safety for residents and tourists (San Martín Gutiérrez and Rodríguez del Bosque, 2010), clean and neat tourist areas (Luque et al. 2007), and spaces that are free from environmental contamination (Echtner, 1991).

### Collaborative process with touristic authorities.

To recover the image of the local and regional destination authorities, inclusive policies and redistributive norms of wealth should be introduced through the social, economic and environmental support of companies in order to maintain local tourism (Jamal et al., 2013). In order to manage cooperatively touristic resources, empowering communities, companies and local associations (Scheyvens 2003) is key.

Local and regional tourist destinations should be built over ethics, fair, equitable and collaborative strategies to delineate the future of the touristic sector. It may involve the setting up of governance models (trusts, consortia, tourist boards, clusters) aimed at sustainable models of tourism cooperation to properly adapt to new tourist scenarios.

Moral strategies based on fair tourism need to be deployed by including both and tourists in designing hierarchic policies. Those would affect not only the way to manage tourist facilities and transport infrastructure but also the provision of emergency health services. Among those moral strategies, the following should be included 1.) the criteria of mere economic gain should be replaced by criteria of cooperation in the governance of tourism resources to rebuild the tourism industry, following the example given by citizens in the health sector and the involvement of civil authorities (Ezebilo & Mattsson, 2010), 2.) prioritizing the local participation of companies, communities and associations in decision-making essentially contributes to the promotion of local interests (Scheyvens, 2003) and this same attitude must be transferred to mitigate the devastating consequences that the health crisis is causing tourism (Moreno-Luna et al., 2021) health authorities should not only emphasize local and regional regulations to benefit tourism companies, but also communities by training local companies and local communities to tackle the power frequently manifested by large corporations outside their territories (Lucchetti & Font, 2013), 4.) qualitative development goals may become more relevant if compared to quantitative goals by fairly treating tourism under the umbrella of social, economic and ecological justice (Ashley et al., 2000) and this crisis shall encourage us to choose sustainable tourism over individualistic systems of governance, and 5.) responsible and sustainable approaches are not enough to eliminate ongoing exploitations and injustices in tourism. Higging-Desbiolles, (2008, 359) propose a humanist globalization based on what he called “justice tourism.” It is a radical break from what has come before. Both tourists and tourism business should support need and common societal interest so as to “socialize tourism” (Higging-Desbiolles, 2020, 9). It is a call for local corporation rather than liberal corporations in the design of tourism strategies in the local territories. Humanism globalization is translated into developing alternative models of tourism management such as cooperatives, social enterprises and other forms of social business, promote inclusive education in social tourism, empowering prioritizing workers if tourism business goes bankrupt and secure worker´s right and good working conditions.

Two limitations can be highlighted. First, the lack of similar empirical studies does not allow us to carry out comparative studies in other territories at the local, regional or international level. Second, the limited collaboration of regional authorities in providing information on studies carried out in the area of Cáceres province, where Sierra de Fuentes is located.

As future lines of research, the research team will begin similar studies on the border with Portugal to analyze the effects of COVID-19 and then compare the results to identify new ways of dealing with the pandemic crisis and new approaches to the tourism sector in general.
